# Supplementary material for: MetaRibo-Seq measures translation in microbiomes
Source: Nat Commun. 2020 Jun 29;11:3268. doi: 10.1038/s41467-020-17081-z (PMC7324362; doi:10.1038/s41467-020-17081-z)
Supplement: Supplementary file 10 — Supplementary Data 7 [file 41467_2020_17081_MOESM10_ESM.zip › File2/Confidence_High_Taxonomy/386997_out.krona.html]

Javascript must be enabled to view this page.

members
magnitude
magnitudeUnassigned
count
unassigned
taxon
rank

386997\_out

15

superkingdom
15
2

phylum
15
976

200643
15
class

171549
15
order

family
171552
14

genus
14
838

species
4

SRS012969\_contig\_number\_29450SRS015794\_contig\_number\_9892SRS077849\_contig\_number\_22048SRS144537\_contig\_number\_47476
2292365

species
165179
10

SRS012849\_contig\_number\_21646SRS012969\_contig\_number\_18520SRS014415\_contig\_number\_1351SRS019285\_contig\_number\_64SRS019808\_contig\_number\_14383SRS045713\_contig\_number\_contig-100\_1128.124358SRS1041134\_contig\_number\_contig-100\_1218.68363SRS1041140\_contig\_number\_22664SRS104912\_contig\_number\_17614SRS143181\_contig\_number\_1647

family
1
815

1
816
genus


SRS146764\_contig\_number\_24427
1
671267
species
